# Autonomous Development of Active Binocular and Motion Vision Through Active Efficient Coding

**DOI:** 10.3389/fnbot.2019.00049

**Published:** 2019-07-16

**Authors:** Alexander Lelais, Jonas Mahn, Vikram Narayan, Chong Zhang, Bertram E. Shi, Jochen Triesch

**Affiliations:** ^1^Frankfurt Institute for Advanced Studies, Frankfurt, Germany; ^2^Department of Electronic and Computer Engineering, Hong Kong University of Science and Technology, Kowloon, Hong Kong

**Keywords:** autonomous learning, active perception, binocular vision, optokinetic nystagmus, smooth pursuit, efficient coding, intrinsic motivation

## Abstract

We present a model for the autonomous and simultaneous learning of active binocular and motion vision. The model is based on the Active Efficient Coding (AEC) framework, a recent generalization of classic efficient coding theories to active perception. The model learns how to efficiently encode the incoming visual signals generated by an object moving in 3-D through sparse coding. Simultaneously, it learns how to produce eye movements that further improve the efficiency of the sensory coding. This learning is driven by an intrinsic motivation to maximize the system's coding efficiency. We test our approach on the humanoid robot iCub using simulations. The model demonstrates self-calibration of accurate object fixation and tracking of moving objects. Our results show that the model keeps improving until it hits physical constraints such as camera or motor resolution, or limits on its internal coding capacity. Furthermore, we show that the emerging sensory tuning properties are in line with results on disparity, motion, and motion-in-depth tuning in the visual cortex of mammals. The model suggests that vergence and tracking eye movements can be viewed as fundamentally having the same objective of maximizing the coding efficiency of the visual system and that they can be learned and calibrated jointly through AEC.

## 1. Introduction

The development of sensorimotor and cognitive skills in humans and other animals provides a rich source of inspiration for research in robotics and artificial intelligence. For example, how can we build robots that acquire intelligent behavior in an autonomous and open-ended developmental process mimicking that of human infants? And, in turn, can we use such robotic models to better understand the computational principles underlying human development?

Early stages of human development are largely concerned with learning to control various sensorimotor systems. These systems form the foundation for the later development of higher cognitive functions. Specifically, some of the earliest sensorimotor skills developing in human infants are related to active visual perception. The infant needs to make sense of the signals arriving at her eyes and she needs to learn how to move her eyes to facilitate perception of the world around her. For the development of visual representations (in particular early visual representations) the Efficient Coding Hypothesis has been the most influential theory. Inspired by the development of information theory, Attneave (1954) and Barlow (1961) have argued in their pioneering works that the visual system exploits the statistical regularities of visual input in order to encode the visual scene efficiently. Furthermore, Barlow (1961) conjectured that early sensory systems have evolved to maximize the amount of information about the visual scene passed to successive processing stages with a constraint of minimizing the associated metabolic costs. Later, the work of Olshausen and Field (1996) established a relation between the statistical structure of natural images and the response properties of cortical simple cells. They proposed to represent natural image patches as linear combinations of sparsely activated basis functions in order to encode the regularities in the images efficiently. Their experiments revealed that a model which learns sparse codes of natural scenes succeeds in developing receptive fields similar to those in the visual cortex. Since then, many experiments have supported the idea that efficient coding is a ubiquitous strategy employed in multiple modalities across diverse organisms (Olshausen and Field, 2004).

A recent extension of the efficient coding hypothesis is Active Efficient Coding (AEC). AEC postulates that biological sensory systems do not just seek to encode the sensory input efficiently, but that they also utilize motor behaviors, such as eye movements, to further improve their coding efficiency (Zhao et al., 2012; Lonini et al., 2013b). Thus, AEC studies efficient coding in the context of behavior and considers the full perception-action cycle and how the organism's behavior shapes the statistics of the sensory signals. AEC works by combining a sparse coding model with a reinforcement learner, which is responsible for generating actions. The sparse coding model learns to efficiently encode the visual input, which serves as a state representation for the reinforcement learner. The reinforcement learner generates actions in order to increase the coding efficiency of the sparse coder.

In previous work, we have successfully applied the AEC approach to model the development of disparity tuning and vergence eye movements using both discrete (Zhao et al., 2012; Lonini et al., 2013b) and continuous actions (Klimmasch et al., 2017). In addition, we have shown that the AEC framework can also be used to model the development of other eye movements such as smooth pursuit (Zhang et al., 2014) and the optokinetic nystagmus (Zhang et al., 2016). Furthermore, the approach has been extended to also learn attention shifts via overt saccadic eye movements (Zhu et al., 2017). In the present study, we present an integrated model of the autonomous learning of active depth and 3-D motion perception using the AEC framework. The model autonomously learns to generate vergence and smooth pursuit eye movements in the presence of a stimulus moving in 3-D. Learning is driven by the agent's intrinsic motivation to maximize its coding efficiency. The advancement to our previous work is the integration of learning to perceive and fixate stimuli located in 3-D and to perceive and track the 3-D motion of respective stimuli. Our results show that the model self-calibrates its eye movement control, improving its performance until it either hits a physical constraint (camera or motor resolution) or runs out of internal resources (capacity of the sparse coding model). Thereby we show and explain the limitations of the model. Furthermore, we show that the model's learned representation of the visual input matches recent findings on the tuning properties of neurons in visual cortex coding for 3-D motion. Thus, the model offers an explanation of how these tuning properties develop in biological vision systems.

## 2. Materials and Methods

### 2.1. Model Overview

**Figure 1:**
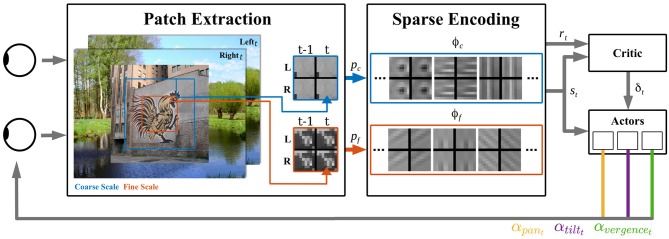
Overview of the active vision architecture. From the binocular visual input at time points *t* − 1 and *t*, patches of different resolutions are extracted for the coarse *p*_*c*_ (blue) and fine *p*_*f*_ (red) scale. These patches are encoded by spatio-temporal basis functions of the coarse scale (blue) and fine scale (red) sparse coders. The activations of both sparse coders' basis functions ϕ_*c*_ and ϕ_*f*_ form the state vector *s*_*t*_. The negative reconstruction error indicates the encoding efficiency and is used as the reward signal *r*_*t*_ for the reinforcement learner. The Critic computes from *r*_*t*_ and *s*_*t*_ a TD-error δ_*t*_ and three distinct actors generate from *s*_*t*_ movement actions α_pan,*t*_, α_tilt,*t*_, α_vergence,*t*_ for the respective camera joints.

Our model consists of three distinct parts (see Figure 1) explained in detail below. At first, one image per camera is preprocessed and dissected into sets of patches. These are encoded by spatio-temporal basis functions of a sparse coding model. This forms a state representation of the sensory input. The state information is processed by a reinforcement learner, which generates camera movements. The negative reconstruction error of the sparse coding stage serves as an indicator of the efficiency of sensory encoding and is used as the reward signal of the reinforcement learner. After execution of the calculated camera movement, the next image pair is sensed and the perception-action cycle starts anew.

### 2.2. Simulation

**Figure 2:**
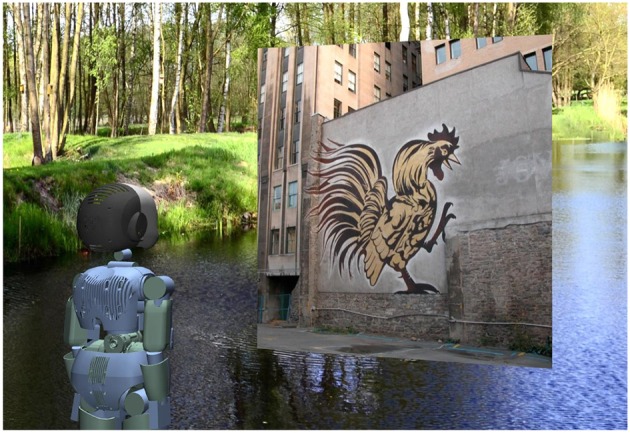
The agent operating the iCub robot inside the virtual environment rendered by the Gazebo simulator.

We simulate the perception-action cycle by using Gazebo^1^, a well known open-source robot simulation platform. Our agent operates the iCub^2^ robot in a rendered virtual environment by moving its cameras (see Figure 2). The two cameras have a horizontal field of view (FOV) of 90° and a resolution of 320 px × 240 px. The distance between the cameras is *d*_*E*_ = 0.068 m. The visual stimuli presented to the agent were taken from the *man made* section of the McGill Calibrated Color Image Database (Olmos and Kingdom, 2004), which contains natural images of urban scenes. Each stimulus had a resolution of 600 px × 600 px. The stimuli were placed on a 1.5 m × 1.5 m plane, perpendicular to the gaze direction. The plane moved within ±30° vertically and horizontally from the agent's center of FOV and [1, 2.5] m in depth. The background image in our virtual environment was taken from Frank Schwichtenberg^3^ and is licensed under CreativeCommons (CC BY-SA 4.0).

### 2.3. Image Processing

At first the images from the left and right camera are gray scaled and convolved with a combined whitening/low-pass filter, a method described by Olshausen and Field (2004). The frequency response of that filter is defined by $R\left(f\right)=fe^{-{\left(\frac{f}{f_{0}}\right)}^{n}}$, where we set the cutoff frequency *f*_0_ = 96 px/image and the steepness parameter *n* = 4. Olshausen and Field (2004) stated that such a filter not only reduces various challenges in operating on digitized natural images but roughly resembles the spatial-frequency response characteristic of retinal ganglion cells. Following Lonini et al. (2013b) we use multiple spatial scales to increase the operating range and robustness of our model. Specifically, we extract two sub-windows with different resolutions from the preprocessed left and right camera image. The first sub-window is a coarse scale 128 px × 128 px image, which covers 36° FOV and corresponds approximately to the human near peripheral vision. To simulate the coarser resolution in the peripheral parts of the human FOV, this image is down-sampled by a factor of 4 via a Gaussian pyramid, resulting in a 32 px × 32 px coarse scale input. The second sub-window is a fine scale 64 px × 64 px image, which covers 18° FOV and corresponds approximately to human macular vision. This image is not down-sampled. Each pixel in the coarse (fine) scale image corresponds to 1.125 (0.281) degrees. Subsequently, patches of 8 px × 8 px size with a stride of 4 px are cut for each scale and camera and normalized to zero mean and unit norm. At each point in time of the simulation, respective patches of the left and right camera image for the current and last iteration are combined to a 16 px × 16 px binocular spatio-temporal patch. This is conducted for each scale and the sets of patches are then encoded by the respective sparse coders.

### 2.4. Sparse Coding

The sensory input is encoded by sparse coding models for the two scales. For each scale *S* ∈ {*c, f*} there is a corresponding dictionary B_*S*_ of spatio-temporal basis functions ϕ_*S, i*_ with |B_*S*_| = 600. For the coarse scale, there are |*p*_*c*_| = 49 spatio-temporal patches and for the fine scale there are |*p*_*f*_| = 225. Each spatio-temporal patch *p*_*S, j*_ is encoded by a linear combination of 10 basis functions ϕ_*S, i*_ to form an approximation ${\hat{p}}_{S,j}$ of the respective patch by

(1) $${\hat{p}}_{S,j}=\sum_{i=1}^{\left|B_{S}\right|}\kappa_{S,i}^{j}\phi_{S,i}\;.$$

This is accomplished by the matching pursuit algorithm (Mallat and Zhang, 1993), where we restrict that $10\;\kappa_{S,i}^{j}$ are non-zero. Hence, we ensure a sparse encoding by using only a subset of 10 basis functions from the dictionary to approximate each image patch. The error of this approximation is the reconstruction error *E*_*S*_ (Lonini et al., 2013b), defined as

(2) $$\begin{array}{l} E_{S}=\overset{\left|p_{S}\right|}{\underset{j=1}{\sum }}\frac{||p_{S,j}-{\hat{p}}_{S,j}|{|}^{2}}{||p_{S,j}|{|}^{2}}. \end{array}$$

We use the negative of the total reconstruction error *E* = *E*_*c*_ + *E*_*f*_ as the reinforcement signal in the next stage of the procedure. The sparse coding model creates as the last step a feature vector *s*_*t*_ of size 2|*B*_*S*_|, which serves as the sensory state information for the reinforcement learner. Every entry in *s*_*t*_ corresponds to the mean squared $\kappa_{S,i}^{j}$ over all patches. This state representation is motivated by the results of Freeman and Ohzawa (1990) who demonstrated that the response of complex cells could be modeled by summing the squared outputs of preceding simple cells. In our case one can interpret the ϕ_*S, i*_ as receptive fields of simple cells in the visual cortex and the entries in the feature vector as activities of complex cells which pool the activities of simple cells over a larger portion of the visual field.

The receptive field (RF) of a neuron in the visual system refers to the visual attributes of a stimulus it is confronted with which generate a response in that cell. The attributes encoded cover a wide range, such as location within the visual field, orientation, disparity, motion direction, velocity and contrast to name a few. Jones and Palmer (1987) have shown that the RFs of neurons in cat striate cortex are particularly well characterized by 2D Gabor filters. The idea that visual input is encoded by elementary components resembling Gabor functions is supported by Bell and Sejnowski (1997). They demonstrated that orthogonal decompositions of natural scenes lead to filters which are best characterized by Gabor-like functions. How RFs arise in living organisms remains a big topic of investigation. In her review of retinal waves Wong (1999) provides support that these patterns of coordinated activity of the premature retina mediate the shaping of structure and function of the visual system in animals already before birth. The current point of view is that the foundations of the visual system are established by spontaneous activity and molecular cues before eye opening (Huberman et al., 2008; Hagihara et al., 2015). Subsequently, the system is fine tuned by visual experience, especially in the so called *critical period* of development (Thompson et al., 2017). Chino et al. (1997) have quantified the fine tuning of response properties of disparity selective V1 neurons in macaque monkeys during the first four postnatal weeks. They found that a coarse disparity selectivity was already present at the sixth postnatal day. In recent studies it has been shown that RF properties such as orientation and direction sensitivity are even established in mice when they are dark-reared (Ko et al., 2014). In view of this background we initialize our basis functions as Gabor wavelets. Specifically, we assume that neurons in the visual system have RFs resembling 2D Gabor functions already before visual experience is gained. However, we do not assume any correlations in time representation of pairs of RFs or space representations of left and right eye encoding RFs. Thus, we initialize each of the four sub-fields of all ϕ_*S, i*_ with independent random 2D Gabor functions, defined by

(3) $$\begin{array}{l} g{\left(x,y\right)}_{\Delta }=\exp\left(-\frac{{x\prime }^{2}+\beta^{2}{y\prime }^{2}}{2\sigma^{2}}\right)\cos\left(2\pi \frac{x^{\prime }}{\lambda }+\psi \right) \end{array}$$

(4) $$\begin{array}{l} \Delta =\left\{\lambda ,\theta ,\psi ,\sigma ,\beta ,x_{c},y_{c}\right\} \end{array}$$

(5) $$\begin{array}{l} x^{\prime }=\left(x-x_{c}\right)\cos\theta +\left(y-y_{c}\right)\sin\theta \end{array}$$

(6) $$\begin{array}{l} y^{\prime }=-\left(x-x_{c}\right)\sin\theta +\left(y-y_{c}\right)\cos\theta , \end{array}$$

where λ is the wavelength of the sinusoidal factor, θ represents the orientation, ψ is the phase offset, σ is the standard deviation of the Gaussian envelope, β is the spatial aspect ratio which specifies the ellipticity, and *x*_*c*_, *y*_*c*_ are the coordinates of the center. The parameters were drawn from uniform distributions over the following intervals: $\lambda \sim \left[\frac{8}{3},16\right]\text{px}$, θ~[0, 180]deg, ψ~[0, 360]deg, $x_{c},y_{c}\sim \left[\frac{8}{3},8\right]\text{px}$. The aspect ratio of the Gaussian envelope was set to $\beta =\frac{\lambda }{0.8\cdot 8}\text{px}$ and the envelope's standard deviation was kept constant σ = 2.5px.

The basis functions are adapted during the training to represent the visual input in the best way with respect to its reconstruction. Therefore, the basis functions are updated through gradient descent on the reconstruction error (Olshausen and Field, 1996):

(7) $$\begin{array}{l} \Delta \phi_{S}=\eta \kappa_{S}\left(p_{S}-{\hat{p}}_{S}\right)\frac{1}{\left|p_{S}\right|}, \end{array}$$

where η is the learning rate, which we set to 0.5 for both scales. After each update step all basis functions are normalized by their energy.

### 2.5. Reinforcement Learning

In the course of training our agent learns to use the sensory state representation to generate camera movements. For this we use a reinforcement learning approach (Sutton and Barto, 1998) named natural-gradient actor-critic (NAC) with advantage parameters (Bhatnagar et al., 2009). The critic learns to approximate the value function given the current state *s*_*t*_, which is represented by the sensory state vector provided by the sparse coding model. The actor is generating movement commands on the basis of the current state, which results in a new state and a reward. The goal of the reinforcement learning is to select actions which maximize the discounted cumulative future reward, defined by $R\left(t\right)=\overset{\infty }{\underset{i=0}{\sum }}\gamma^{i}r_{t+i}$, where we set the reward *r*_*t*_ = −*E*_*t*_ and the discount factor γ = 0.3. The value function is learned by computing the temporal difference (TD) error δ_*t*_ and approximating the average reward Ĵ_*t*_. The TD-error is defined by Equation 8, where ${\hat{V}}_{t}\left(s_{t}\right)=\langle \theta_{t}^{V},s_{t}\rangle$ is the critic's current value function approximation with $\theta_{t}^{V}$ being the respective parameter vector and 〈, 〉 indicating the inner product of two vectors. The approximation of Ĵ_*t*_ is defined by Equation 9 which is equivalent to low-pass filtering *r*_*t*_, where ξ = 0.01 is the smoothing factor. For the value function approximation we use a two layer artificial neural network (ANN) with |*s*_*t*_| input neurons, one output neuron and θ^*V*^ as weights between the layers. The weights are updated by Equation 10, where α = 0.4 is the learning rate of the critic. The low value of γ was found empirically to produce good performance. As the agent receives a reward in every iteration there is no issue of delayed rewards and therefore a fairly strong discounting of future rewards does not disadvantage the learning or performance.

(8) $$\begin{array}{l} \delta_{t}=r_{t}-{Ĵ}_{t}+\gamma {\hat{V}}_{t}\left(s_{t}\right)-{\hat{V}}_{t-2}\left(s_{t-2}\right) \end{array}$$

(9) $$\begin{array}{l} {Ĵ}_{t}=\left(1-\xi \right){Ĵ}_{t-1}+\xi r_{t} \end{array}$$

(10) $$\begin{array}{l} \Delta \theta_{i,t}^{V}=\alpha \delta_{t}s_{t-2} \end{array}$$

The movement commands are generated by three individual actors which control the agent's pan, tilt, and vergence joints of the cameras, respectively. Each actor maps the current *s*_*t*_ to an action *a* ∈ A = {−16, −8, −4, −2, −1, −0.5, 0, 0.5, 1, 2, 4, 8, 16}. The actions of the pan and tilt joint controlling actors are interpreted as acceleration commands of the cameras, whereas the vergence joint controlling actor's output is interpreted as change in the vergence angle of the cameras. Therefore, the units for the pan and tilt actions are deg/s^2^ and deg for the vergence actions. Each actor is implemented as a two layer ANN with |*s*_*t*_| input neurons, |A| output neurons and θ^*A*^ as weights between the layers. The activation *z*_*a, t*_ of the output neuron corresponding to the respective action *a* is computed by $z_{a,t}=\langle \theta_{a,t}^{A},s_{t}\rangle$. The actions applied are chosen by sampling from a Softmax policy, where the probability π_*a, t*_ for action *a* is

(11) $$\pi_{a,t}=\frac{\exp(z_{a,t}T^{-1})}{\sum_{n=1}^{\left|A\right|}\exp(z_{n,t}T^{-1})}\;,$$

where *T* is the temperature parameter, which controls the exploration vs. exploitation behavior of the agent. We set *T* = 1 to ensure the agent explores while learning. The actors' weights θ^*A*^ are updated by

(12) $$\begin{array}{l} \zeta_{t}=\nabla_{\theta }\log\pi_{\theta }\left(a_{t-2}|s_{t-2}\right), \end{array}$$

(13) $$\begin{array}{l} \Delta w_{t}=\beta \left(\delta_{t}\zeta_{t}-\zeta_{t}\left(\zeta_{t}^{T}w_{t-1}\right)\right), \end{array}$$

(14) $$\begin{array}{l} \Delta \theta_{t}^{A}=\eta w_{t}, \end{array}$$

where ζ_*t*_ are the policy derivatives, *w*_*t*_ are the advantage parameters, β is the learning rate of the natural gradient and η is the learning rate of the actor. The family of NAC algorithms are reinforcement learning approaches, which combine learning from the TD-error δ_*t*_ and a policy gradient. However, instead of following the regular (vanilla) policy gradient, NAC algorithms are following the *natural* gradient to update the actor's weights θ^*A*^. A thorough derivation and discussion of the natural gradient is provided, e.g., by Peters et al. (2005). The NAC algorithm with advantage parameters *w*_*t*_ does not explicitly store an estimate of the inverse Fisher information matrix, which the other members of the NAC family are using to follow the natural gradient as Bhatnagar et al. (2009) point out. This makes the NAC algorithm with advantage parameters computationally cheaper and the approximation of the natural gradient through the *w*_*t*_ is comparable to the other members of the NAC family. The interested reader is referred to Bhatnagar et al. (2009) for derivations of Equations 8–10 and Equations 12–14 and convergence analysis and discussion of various NAC algorithms. We set for all actors β = 0.16 and η = 0.4. Due to the model's architecture, it takes two iteration steps until an action has its full effect on the state representation. Therefore, we update the critic and the actors with respect to *s*_*t*−2_ and *a*_*t*−2_.

### 2.6. Experimental Procedure

In our experiments we probe the agent's capability to learn to fixate and track a moving stimulus. Each experiment consists of 5·10^5^ training iterations, each corresponding to 100 ms. Experiments are repeated 10 times with different randomization seeds. Training is divided into intervals, each lasting 40 iterations. At the start of each interval, a stimulus is drawn at random from a set of 100 images from the McGill dataset and centered in the agent's FOV. The stimulus is positioned at a distance to the agent drawn from a uniform distribution over [1, 2.5] m. The agent initially fixates on a point directly in front of it at a distance chosen at random from the interval [0.3, 3] m. During the interval, the stimulus moves according to velocities drawn from uniform distributions over [−7.5, 7.5]deg/s in the horizontal and vertical directions and [−0.375, 0.375]m/s in depth. The agent updates the pan and tilt velocities of its eyes and the vergence angle between them according to the policy. In case any joint exceeds a pre-defined angle boundary (±15deg for the pan/tilt joint, [0.2, 16.3]deg for the vergence joint), the joint velocities are set to zero and the agent's gaze is reset as described afore.

## 3. Results

**Figure 3:**
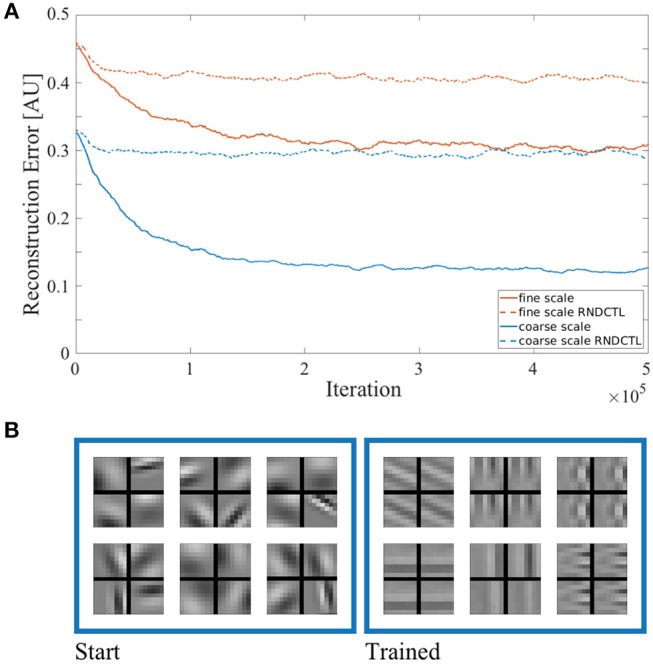
**(A)** Reconstruction error of the sparse coding model. The error is plotted in arbitrary units vs. training time for the coarse scale (blue) and fine scale (red) sparse coder in solid lines. The model's encoding performance in a control experiment, where the actions were uniformly sampled at random from the same action sets (RNDCTL) is plotted in dashed lines. **(B)** Coarse scale basis functions. Six representative spatio-temporal basis functions of a coarse scale dictionary are shown at the start (left) and the end of training (right). Every basis function consists of 4 parts. The rows show the corresponding patch for the left (top) and right (bottom) eye. The columns represent the patch for time *t* − 1 (left) and *t* (right).

We start by presenting the quality of sensory state encoding of our approach. Figure 3A shows the reconstruction error of both sparse coders vs. training time in solid lines. The improvement of stimulus reconstruction in both scales over the course of training clearly shows an increase in coding efficiency. As we enforce the encoding to be sparse (see Equation 1), the agent works with the same small amount of resources throughout training. Hence, by improving the encoding result using the same amount of resources as at the start of training, the agent increases its encoding efficiency. We also tested the encoding performance of the sparse coding model in a testing procedure (which is described further below) with a stimulus set disjoint from the training set. As the agent showed similar reconstruction capabilities in both training and testing procedures (compare Figure S3), the learned sparse coding dictionary can be considered generic (at least for urban scenes and man-made objects as they occur in the data base). In a control experiment we used the same model but exchanged the action generation of the reinforcement learning (RL) by a uniform sampling at random of the pan, tilt and vergence actions from the same action sets we used before. The encoding performance of both sparse coders in this control experiment is shown in Figure 3A in dashed lines. The sparse coders' coding efficiency does not significantly improve in this setup in the course of training compared to the model using RL for action generation. This shows that the RL does improve the coding efficiency in our AEC framework. In Figure 3B six representative spatio-temporal basis functions of the coarse scale dictionary are depicted at initialization time and at the end of the training. The fine scale bases look similar. The basis functions were initialized by random Gabor wavelets, but the sparse coding model has adjusted the bases to properly encode the stimulus statistics it was confronted with.

**Figure 4:**
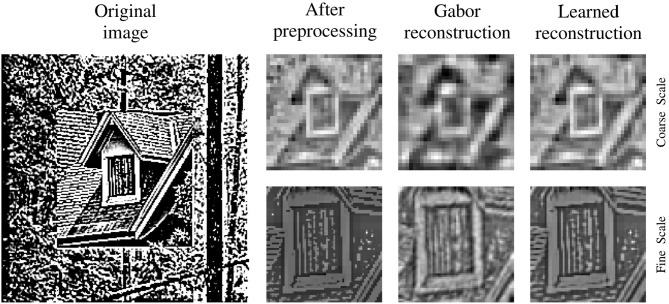
Input image reconstruction. Depicted are column wise from left to right the original whitened camera image, the cropped, down-sampled, and normalized input image for the coarse scale (top row) and fine scale (bottom row) sparse coders. Right to the preprocessed images are the respective images reconstructed with random Gabor wavelets at initialization time and the images reconstructed with learned basis functions at the end of training.

For a qualitative impression of the reconstruction performance, a stimulus is shown at different processing and training stages in Figure 4. The comparison of the preprocessed input images and the respective reconstructions thereof, shows a clear improvement of the reconstruction quality between the sparse coding model at initialization time and at the end of training. All images are shown for the left eye and its respective basis parts at time *t* are used for encoding and reconstruction. For a fair comparison between the trained and the untrained agent the joint angles of the cameras are set to perfectly fixate the center of the stimulus. The image reconstruction is already at initialization time fairly decent due to the size of the sparse coding dictionary, the amount of basis functions used for individual patch encoding and the perfect fixation of the stimulus. Though, the encoding and therefore the reconstruction improves as the basis functions are adapted to the stimulus statistics. The image reconstruction with white noise initialized basis functions looks more noisy at initialization time but similar at the end of training (see Supplementary Material).

**Figure 5:**
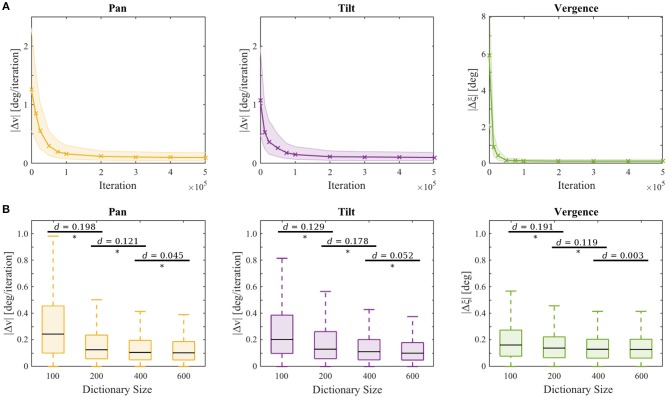
**(A)** Testing performance vs. training iteration. Depicted are the respective errors in the pan Δ*v* (yellow), tilt Δ*v* (purple), and vergence Δξ (green) joint of the testing procedures for all test stimuli and movement speeds over 10 trials at the respective points in time during the training procedure. The lines represent the median errors and the shaded areas show one inter quartile range. **(B)** Testing performance at the end of training for agents with different sizes of sparse coding dictionaries over 3 experiment repetitions. Significant differences (*p* < 0.05) between two sets of data are assessed by a *t*-test and marked (*). Horizontal bars indicate effect size as measured by Cohen's *d*.

We tested the policy at 10 points during training for 50·6^3^ = 10800 trials, each corresponding to one of the possible combinations of 50 stimuli chosen from a set of images from the McGill database disjoint from the training set and 6 velocities in each of the three directions (horizontal, vertical and depth). The velocities were chosen from {±0.1, ±0.5, ±1} times the maximum velocities in each direction. Each trial lasted for 10 iterations, as no performance improvement was gained after that. To correctly track the stimulus, the agent needs to rotate its eyes with the same speed as the stimulus is moving in the respective direction. Therefore, the errors for the pan and tilt joints Δ*v* were measured in deg/iteration as the difference between the speeds of the object and the eyes at the last iteration of the trial. The error for the vergence joint Δξ was defined as the difference between the actual and desired vergence angle, which was computed by

(15) $$\begin{array}{l} \xi^{*}=2\arctan\left(\frac{d_{E}}{2d_{O}}\right), \end{array}$$

where *d*_*E*_ is the horizontal separation between the eyes and *d*_*O*_ is the object distance. During the performance assessment, the learning of the sparse coders and the reinforcement learner was switched off and the actors applied a greedy policy. The testing performance is depicted in Figure 5A. For each of the respective joints the median of the absolute error at the last iteration of a testing trial is plotted in solid lines and one IQR is indicated by shading. Statistics are computed over all testing trials.

We also examined the influence of the sparse coder's basis function dictionary size on the testing performance. Figure 5B shows the testing performance after training for 5·10^5^ iterations for |B_*S*_| ∈ {100, 200, 400, 600} on the same test stimulus set used before. Each experiment was repeated 3 times with different randomization seeds. A student's *t*-test revealed a significant improvement (*p*-values < 10^−8^) for all comparisons marked in Figure 5B. The errors decrease with increasing dictionary size. Calculation of the effect sizes by Cohen's *d* (Cohen, 1988) showed that increasing the dictionary size to 800 results in a neglectable effect of *d* < 0.045 compared to |B_*S*_| = 600. Therefore, we conclude that the model's performance saturates when ~ 600 basis functions are present. Initializing the basis functions with white noise yielded similar results (see Supplementary Material), though the learning progress was less robust, as the IQRs were bigger before convergence.

**Figure 6:**
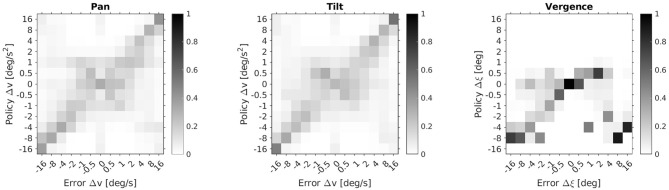
Learned policy distributions averaged over 10 agents and 50 stimuli. Depicted are action probabilities for the respective pan, tilt and vergence actor as a function of state errors.

In Figure 6 we provide a more detailed view of the learned policies averaged over 10 agents and the 50 stimuli of the test set. It shows the probability distributions of the action sets of the respective pan, tilt and vergence actor over a range of errors in the corresponding state space. The ideal policy π^*^ is a diagonal in each case. The pan and tilt actor's policy was probed by moving the stimulus only along the respective dimension. For the vergence actor the stimulus's distance was varied but the object remained static. Thereby, we avoided any interference between the actors. The pan and tilt actors perform more accurately the bigger the absolute speed errors are. For small speed errors the ideal action is not uniquely identified. The vergence policy shows the desired diagonal structure only for negative and small positive vergence errors Δξ. This is due to the ranges of initial eye fixations and stimulus depths in our experimental setup. Specifically, the agent is rarely confronted with big positive vergence errors and never with Δξ > 3 deg (see Equation 15). An accurate vergence policy for large positive Δξ would require a training setup where such vergence errors are encountered regularly.

**Figure 7:**
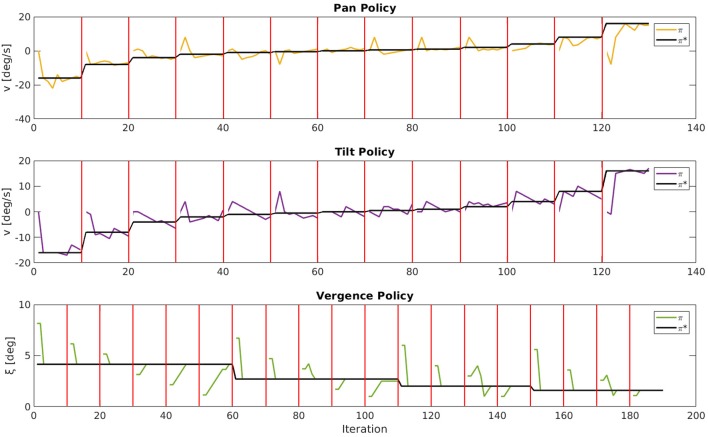
Movement trajectories of an agent for one stimulus. For pan and tilt the respective joint speed was reset to 0 deg/s every 10 iterations as indicated by the red bars. For the vergence joint the fixation angle ξ was initialized with varying vergence errors every 10 iterations. The actual policy π is plotted, respectively, in yellow (pan), purple (tilt), and green (vergence) and the desired policy π^*^ in black.

For a qualitative impression of the behavior we show in Figure 7 good examples of movement trajectories of an agent for one stimulus. For the pan and tilt dimension the stimulus speed changed and the respective joint speed was reset to 0 deg/s every 10 iterations. For the vergence dimension the eyes were initialized with varying Δξ errors every 10 iterations. We demonstrate the agent's performance additionally in a video (see Supplementary Material) for various stimuli and movement speeds. The object tracking is driven by the movement of the object, as the agent can best encode the visual input stream of the moving object if the object remains static on the retina (camera images). The agent manages to establish a static retinal image by moving its eyes with the same speed as the fixated object moves.

**Figure 8:**
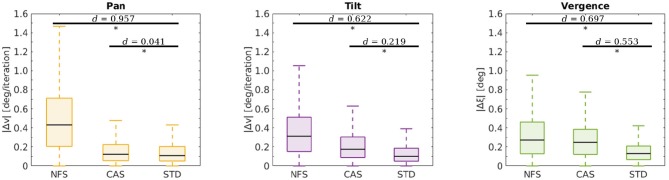
Testing performance at the end of training for agents with different configurations. Depicted are the respective errors in the pan Δ*v* (yellow), tilt Δ*v* (purple) and vergence Δξ (green) joint for all test stimuli and movement speeds. The configurations span the situations when there is no fine scale sparse coder (NFS), a coarser action set (CAS), and when the standard configuration is used (STD). Horizontal bars indicate comparisons between two sets of data as assessed by a *t*-test. Significant differences (*p*-values < 0.05) are marked (*) and effect sizes are indicated as measured by Cohen's *d*.

In two additional experiments we investigated the limits of our model. Both were conducted the same way as described before. In the first experiment we reduced the camera resolution by providing no fine scale sparse coder (NFS) to the agent. In the second experiment we reduced the motor resolution by providing a coarser action set (CAS) to the agent. The coarser action set was defined by A = {−16, −8, −4, −2, −1, 0, 1, 2, 4, 8, 16} for all actors, i.e., the actions ±0.5 have been removed. Figure 8 shows the results of this analysis. A student's *t*-test was used to compare the performance between the agents with NFS and CAS and an agent with standard configuration (STD). The difference between NFS and STD was significant (*p*-values < 10^−57^) with an effect size of Cohen's *d* > 0.622 for all joints. The comparison between the agent with the CAS and STD showed significant differences for the tilt and vergence actor (*p*-values < 10^−57^) with effect sizes of *d* > 0.219. The difference between the pan actors was also significant (*p* = 0.003), but the effect size of *d* = 0.041 was relatively small. These results demonstrate that the agent keeps improving until it hits physical constraints such as camera or motor resolution, or limits on its internal coding capacity as shown in Figure 5B.

### 3.1. Analysis of Basis Function Properties

**Figure 9:**
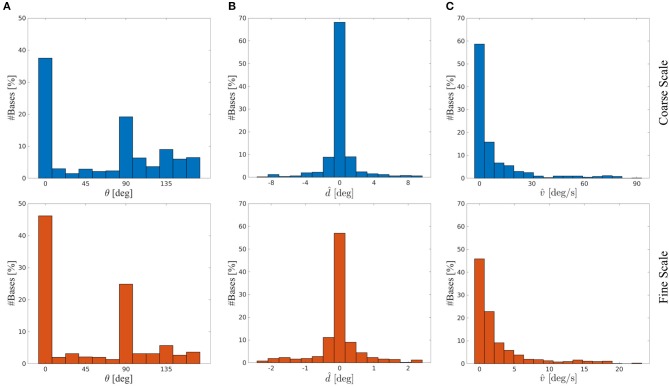
Basis functions' stimulus preferences for the coarse scale (blue) and fine scale (red) from a typical experiment. **(A)** Histogram of orientation preferences θ. **(B)** Histogram of disparity preferences $\hat{d}$. **(C)** Histogram of velocity preferences $\hat{v}$.

We investigated whether the learned basis functions maintained a Gabor-like structure and compared their properties to biological data. For that we fitted 2D Gabor functions (see Equations 3–6) to the four sub-fields of the basis functions. The squared norm of the residual of the basis functions *r* had a mean of μ = 0.003 ± 0.006 *SD* at initialization time. After training the mean of *r* was μ = 0.038 ± 0.034 *SD* for the coarse scale and μ = 0.011 ± 0.016 *SD* for the fine scale basis functions. Basis functions initialized with white noise have a mean of *r* of μ = 0.188 ± 0.022 *SD*. Hence, the basis functions remained Gabor-like. The histograms of orientation preferences θ of the coarse scale (blue) and fine scale (red) basis functions are depicted in Figure 9A. Vertical (~ 42%) and horizontal (~ 22%) orientations are most common. This is in line with biological findings on the so-called *oblique effect*, which show an over-representation of vertical and horizontal RFs in many species such as cats, monkeys and humans (Appelle, 1972; Li et al., 2003). This bias is strongly shaped by the stimulus statistics the agent is facing during training, as there is a prevalence of horizontal and vertical edges. We have investigated RF properties which arise from normal and abnormal rearing conditions in more detail in Klimmasch et al. (2018).

We further analyze the disparity preferences $\hat{d}$ of the basis functions for the respective basis sub-parts representing time *t* for both scales (see Figure 9B). The disparity preference at time *t* is computed by

(16) $$\begin{array}{l} \hat{d}=\frac{\lambda \left(\psi_{t,\text{left}}-\psi_{t,\text{right}}\right)}{2\pi \cos\theta }. \end{array}$$

The distribution of preferred disparities is centered at zero degrees and covers a range of about ±2° for the fine scale and ±8° for the coarse scale. This is consistent with the biological finding that the majority of receptive fields in macaque V1 and middle temporal (MT) visual cortex are tuned to near zero disparities (Prince et al., 2002; DeAngelis and Uka, 2003).

The velocity preference $\hat{v}$ for a given eye, say, the left eye, is computed by

(17) $$\begin{array}{l} \hat{v}=\frac{\lambda |\psi_{t,\text{left}}-\psi_{t-1,\text{left}}|}{2\pi }. \end{array}$$

Figure 9C shows that the basis functions have a preference for encoding low velocities at both coarse and fine scale. Orban et al. (1986) analyzed the velocity preference of V1 and V2 neurons in macaque monkeys and Felleman and Kaas (1984) have shown for the further visual processing path in cortex of owl and macaque monkeys that neurons in the MT cortex are also encoding stimulus velocities but typically higher velocities than neurons in V1 and V2. This is most likely due to the increased receptive field size of MT neurons compared to RF sizes of neurons in lower areas. The stimulus selectivity of our basis function sub-parts show similar $\hat{v}$ distributions to V1 and V2 velocity preference of neurons encoding the central visual field (compared to Orban et al., 1986). Therefore, our results provide support for interpreting the *sub-parts* of our basis functions, i.e., the columns, as RFs of binocular simple cells in V1/V2 and a *complete* basis as the response of a complex cell pooling activities from multiple simple cells.

**Figure 10:**
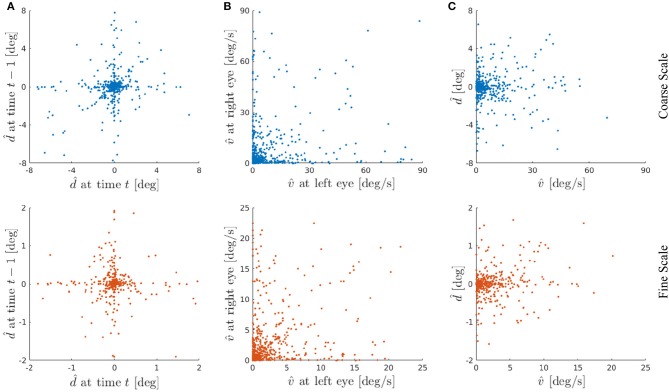
**(A)** Basis functions' disparity preference $\hat{d}$ at time *t* vs. *t* − 1 from a typical experiment. Each dot represents one basis function of the coarse scale (blue) or the fine scale (red). **(B)** Basis functions' velocity preference $\hat{v}$ at left eye vs. right eye. **(C)** Basis functions' velocity $\hat{v}$ vs. disparity $\hat{d}$ preference averaged over left and right eye and time *t* and *t* − 1, respectively. Here we show the basis functions' joint encoding of velocity and disparity. The basis functions are sensitive to a wide range of combinations of preferred velocities and preferred disparities. The velocity and disparity preferences of basis functions are not correlated.

Figure 10A shows the disparity preference $\hat{d}$ of the basis functions at time *t* vs. *t* − 1. This illustrates that the agent has learned representations for all situations it was confronted with during the training phase. Specifically, 45.5 % of the basis functions are representing a disparity of $|\hat{d}|\leq \text{1.125}\;\text{deg}\hat{=}\text{1}\;\text{px}$ in the coarse scale and 40 % of the basis functions encode $|\hat{d}|\leq \text{0.281}\;\text{deg}\hat{=}\text{1}\;\text{px}$ in the fine scale, respectively. These basis functions represent the situations where the agent was fixating the stimulus within 1 px accuracy at time *t* − 1 and kept on fixating it at time *t*. Other basis functions show, e.g., tuning for close to zero disparity at time *t* − 1 but not at time *t*. Such basis functions can detect object movement in depth, where the object leaves the current fixation plane.

In general, various kinds of motion can be encoded with our basis functions, such as fronto-parallel and 3-D motion. In case of equal velocity representation for left and right eye, a fronto-parallel motion is encoded. Whereas different velocity preferences in both eyes represent a motion in depth (Czuba et al., 2014). Figure 10B depicts the results of this analysis for both scales. The linear correlation between the basis parts representing the left and right eye shows a correlation coefficient of ρ = 0.215 for the coarse and ρ = 0.289 for the fine scale. This indicates that most basis functions are encoding motion in depth, nevertheless a considerable amount of basis functions are representing fronto-parallel motion.

Electrophysiological recordings from neurons in the MT area of macaque visual cortex show that most MT neurons are tuned to both binocular disparity and the direction of stimulus motion, and many MT neurons have their disparity and motion tuning independent of each other (DeAngelis and Newsome, 2004). A more recent study of Sanada and DeAngelis (2014) has shown that about a half of the neurons in macaque MT cortex are selective for the direction of motion in depth with some contribution of disparity cues. In this context we analyzed the average velocity preference of both eyes vs. the average disparity preference for *t* and *t* − 1 in Figure 10C to study the results of joint encoding of both velocity and disparity. It is evident from Figure 10C that the velocity and disparity preferences have no linear correlation and thus they respond to a combination of specific disparity and motion. Despite a peak in near zero velocity and disparity, as already seen in Figure 9, one can clearly observe that the learned basis functions are encoding a wide range of velocities and disparities.

The distribution of preferred disparities in the model (see Figure 9B) has less variance compared to biological data (DeAngelis and Uka, 2003; Sprague et al., 2015). We investigated whether the agent encounters a too narrow range of disparities during training, as the range of object distances is small and the objects are planar textures. Hence, we trained our agent as described before, but at each training interval the stimulus was at random either tilted or slanted by an angle uniformly drawn from ±45deg and the stimulus distance was uniformly drawn from [0.3, 1.5] m. As suspected, this manipulation resulted in a larger variance of the distribution of preferred disparities of the basis functions for both scales. We verified this by applying a Brown-Forsythe test on distributions of preferred disparities trained in the standard and the afore mentioned modified scenario. For both the coarse scale (*p* = 1.39·10^−3^) and the fine scale (*p* = 2.81·10^−2^) the test indicated a significant increase in the variance of the distributions of preferred disparities. The testing performance over 10 repetitions with different randomization seeds in this modified scenario was similar to the standard scenario. Hence, with our approach the agent can encode and track non-fronto-parallel objects as well. The study of Zhu et al. (2017) has demonstrated that within our AEC framework an agent can also learn to fixate 3D objects. In an additional control experiment we tested the standard agent with a sphere-shaped object instead of the fronto-parallel plane and projected the same natural textures on top of the sphere as in the standard testing procedure. In a video (see Supplementary Material) we demonstrate that an agent which was trained with a fronto-parallel plane can also fixate and track a sphere-shaped object.

The shape of the stimulus does not limit our approach, but the size of the stimulus does. The extent of the FOV of the agent (see Seq. 2.3), the amount of patches in the coarse and fine scale, the formalization of the reconstruction error (see Equation 2) and the resulting reward signal determine the minimum size of the stimulus which can still be tracked by our agent. As the agent strives to minimize the total reconstruction error *E*, it is fixating and tracking the image regions that contribute most to *E*. Hence, if the stimulus is covering more of the FOV than the background, it is encoded by more patches of the sparse coders and therefore the stimulus contributes more to *E* than the background does. Hence, if <50 % of *E* is accounted for by the stimulus, the agent will focus on the background instead. Considering the number of patches in the fine and coarse scale regions and their overlap, one can estimate that successful tracking requires that the stimulus covers ~80 % of the area of the fine scale. This means that if the stimulus width is 0.5 m it is not rewarding for the agent to fixate and track it when the distance to the stimulus is ≥ 1 m. We show in a video (see Supplementary Material) the agent's behavior in the discussed situations where it is confronted with a 1.5 m, 1.2 m, and a 0.3 m wide object.

## 4. Discussion

The fixation of an object in depth and its pursuit with the eyes when it moves are two elementary visual capabilities that emerge early during human development. We have demonstrated that Active Efficient Coding is well suited as a model for the joint learning of these two basic visual abilities, which were learned separately in our previous works. Our model learns an efficient representation of depth and motion via sparse coding. In parallel, a reinforcement learning component learns to generate a behavior which facilitates the efficient encoding of the scene by the sparse coding component via an intrinsic motivation for coding efficiency. Thereby the agent simultaneously learns a representation of the visual scene and the fixation and pursuit behavior in a completely autonomous fashion. To the best of our knowledge, the joint learning of both sensory representation and behavior is unique to our approach. For example, the recent approach by Konda and Memisevic (2014) also learns disparity and motion representations, but it does so from a fixed set of training videos via supervised learning and it does not include the learning of any behavior which would change the statistics of the sensory signals. Conversely, the approach by Gibaldi et al. (2015) learns to execute vergence eye movements, but the set of filter banks which are used to process the input images is predefined and does not adapt to the statistics of the visual input. Indeed, the majority of existing models for learning vergence or smooth pursuit have a much narrower focus than our work. Early models detected only specific velocities or disparities (Rashbass and Westheimer, 1961; Krishnan and Stark, 1977). Some works only used synthetic and not natural images (Patel et al., 1997; Gibaldi et al., 2010). The studies of Hoyer and Hyvärinen (2000), Hunter and Hibbard (2015), and Chauhan et al. (2018) used unsupervised approaches to learn binocular disparity selectivity from natural stereoscopic images. In the work of Burge and Geisler (2014) disparity selectivity was learned by optimizing disparity discrimination in natural images. Importantly, the focus of these studies was on learning representations of still images and these models do not learn or produce any behavior and none addresses motion selectivity. Beyeler et al. (2016) show a model how the motion signal from MT cortex could be further processed by medial superior temporal (MSTd) cortex. They present an alternative approach how sparse basis functions, which show similar tuning properties as macaque MSTd neurons, could emerge from MT units through a dimensionality reduction technique. In contrast to our work, their MT units are predefined and the model does not generate any behavior. Other works required the engineering of specific image features, knowledge of the intrinsic parameters of the camera, or a predefined model of object velocity or disparity. In addition, most works on motion vision do not address the issue of binocular vision, because they only consider monocular visual input.

The tasks learned by our model, vergence control and smooth pursuit, are similar to those learned by the model of Zhang et al. (2016), vergence control and the optokinetic nystagmus (OKN). Both smooth pursuit and the OKN are minimizing the retinal slip, but smooth pursuit is associated with smaller targets and more voluntary eye movements. The architectures presented here and in Zhang et al. (2016) are similar in that they show the same sparse coding based perceptual stage and the same reinforcement learner for the vergence commands. However, they differ in the learning of the smooth pursuit/OKN. Here we use reinforcement learning, but Zhang et al. (2016) use Hebbian learning combined with scaffolding by a subcortical pathway. The work here provides a more parsimonious model, but Zhang et al. (2016) is more consistent with the observed developmental interactions between the cortical and subcortical pathways underlying the OKN.

Many experimental studies on binocular disparity tuning in the brain have found evidence suggesting that the primary visual cortex (V1) optimally processes the natural binocular disparity statistics. In this regard, the efficient coding hypothesis conjectures that the disparity tuning of V1 binocular neurons reflects the natural range of disparities (Read and Cumming, 2004; Liu et al., 2008) and that eye movement strategy is such that it minimizes the binocular disparity and motor inefficiency (Tweed, 1997; Schreiber et al., 2001). These findings are consistent with our model.

The work of Yu et al. (2005) has shown that neurons in primary visual cortex exhibit higher coding efficiency when responding to correlated signals compared to uncorrelated ones. Our AEC framework similarly exploits correlations in sensory signals that are generated through its own motor behavior. Specifically, as our model learns vergence eye movements it learns to reduce disparities between the eyes and therefore increases the redundancy between left and right camera input. Similarly, our model increases the redundancy between successive images of its cameras as it learns to perform pursuit eye movements. The agent's actions ultimately result in a more efficient encoding of the visual scene, because the model adapts its basis functions to efficiently exploit the redundancies in the sensory signals that it is creating through its own learned motor behavior.

It has been well established in the neuroscience literature that the RFs in primary visual cortex of certain mammalian species already have a Gabor-like structure before visual experience is gathered, i.e., before eye opening. Therefore, we also initialized the basis functions in our model to already have Gabor shapes at the start of learning. Importantly, however, as seems to be the case in biology, the left and right subfields of the basis functions were statistically independent. In addition to the experiments presented above, we also tested if the model can still learn successfully without such a Gabor initialization of the basis functions. We observed that the model still learns successfully, when the basis functions are initialized as independent Gaussian white noise (see Supplementary Material).

The analysis of the basis functions confirms the findings of Qian (1994) and Smolyanskaya et al. (2013) that disparity and motion tuning are largely independent of each other. Czuba et al. (2014) have shown that MT neurons encode 3-D motion and in this regard we also observe the presence of basis functions which have different velocity preferences between left and right eye, thus being sensitive to 3-D motion. Furthermore, some basis functions are also encoding fronto-parallel movement and overall a broad range of velocities and disparities. Therefore, they resemble the encoding properties of real neurons in the visual system.

To the best of our knowledge, apart from our work (Zhang et al., 2016), research on vergence eye movements and research on pursuit eye movements and the optokinetic nystagmus has been progressing independently. In stark contrast to this tradition, our new model suggests that these phenomena can be unified and seen as special cases of the general idea of Active Efficient Coding, i.e., the idea of a sensory system exploiting its motor degrees of freedom to support the efficient encoding of information from the environment. In fact, recent work suggests that torsional eye movements (Zhu et al., 2018) and the control of accommodation (Triesch et al., 2017) are just two further instances of this very general idea.

In previous studies we have shown that our AEC approach also works on the iCub robot in a real life scenario (Lonini et al., 2013a; Teulière et al., 2015). As our model presented in this study shows good performance on the simulated iCub, we are confident that future studies will prove its robustness on the real iCub. This should be tested in future work.

The present model may also have implications for developmental disorders of the visual system such as strabismus and amblyopia (Eckmann et al., 2018). As a first model of how sensory and motor aspects of binocular and motion vision jointly develop and self-calibrate, it may be a useful testbed for studying what factors can derail this development in developmental disorders and what treatments may bring it back on track.

## Author Contributions

JT, AL, and JM designed the experiments. AL and JM conducted the experiments. VN and CZ implemented initial versions of the model and AL and JM implemented the final version of it. CZ implemented a first version of the Gabor wavelet fitting algorithm and AL and JM implemented the final version of all analysis related algorithms. All authors contributed to the paper writing.

### Conflict of Interest Statement

The authors declare that the research was conducted in the absence of any commercial or financial relationships that could be construed as a potential conflict of interest.

## Footnotes

^1^http://gazebosim.org/

^2^http://www.icub.org/

^3^https://commons.wikimedia.org/wiki/File:Uetersen_Langes_Tannen_Panorama_02.jpg

## Supplementary Material

Video 1 Active binocular and motion vision—testing performance.

Video 2 Active binocular and motion vision—testing with 3D stimuli and limitations.
