# Supplementary material for: Autonomous Development of Active Binocular and Motion Vision Through Active Efficient Coding
Source: Front Neurorobot. 2019 Jul 16;13:49. doi: 10.3389/fnbot.2019.00049 (PMC6646586; doi:10.3389/fnbot.2019.00049)
Supplement: Supplementary file 3 [file Data_Sheet_1.PDF]

Supplementary Material: Autonomous Development of Active Binocular and Motion Vision through Active Efficient Coding

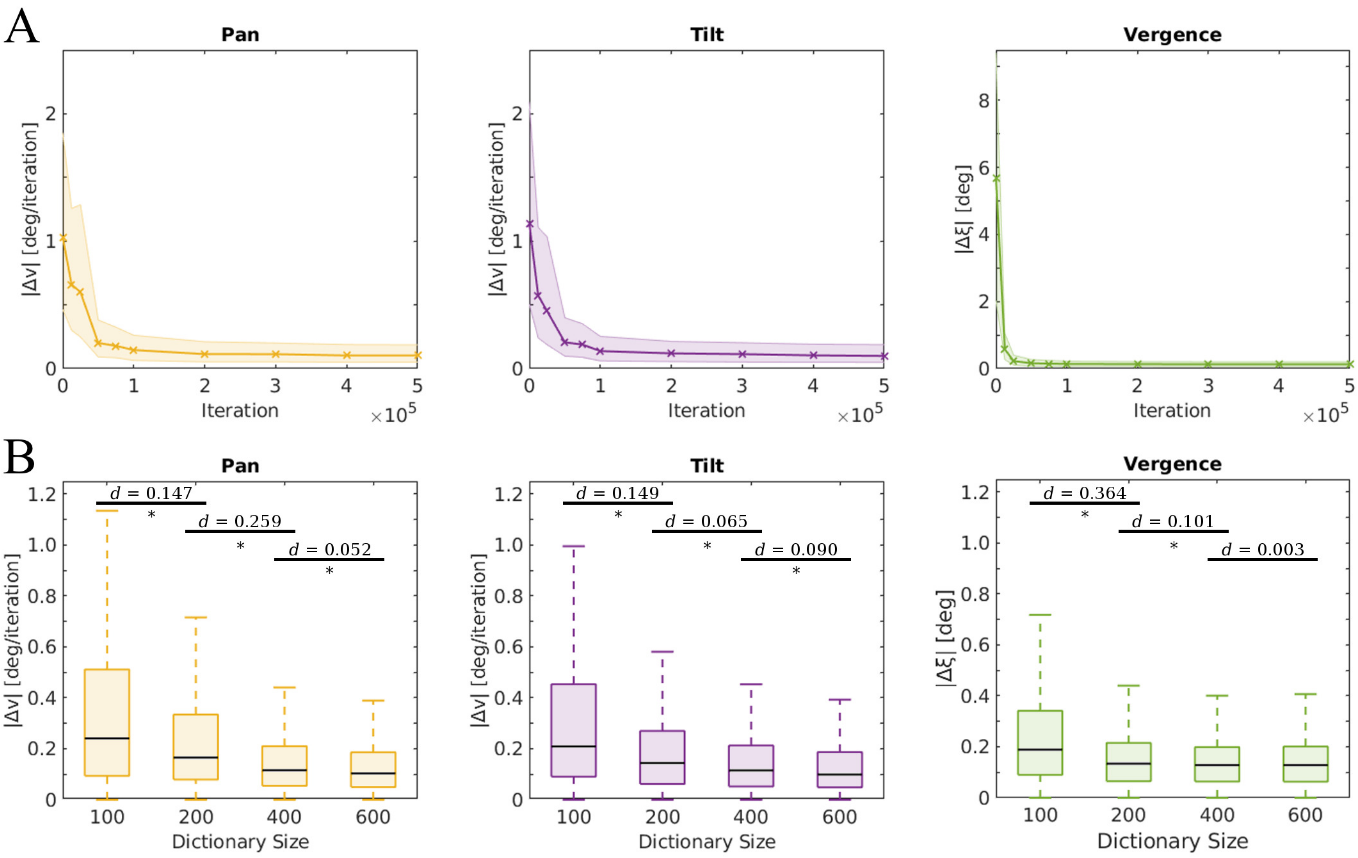

Figure S1. **(A)** Testing performance versus training iteration when the basis functions of the model are initialized with white noise. Depicted are the respective errors in the pan (yellow), tilt (purple) and vergence (green) joint of the testing procedures for all test stimuli and movement speeds over 5 trials at the respective points in time during the training procedure. The lines represent the median errors and the shaded areas show one inter quartile range. **(B)** Testing performance at the end of training for agents with different sizes of sparse coding dictionaries over 5 experiment repetitions. Horizontal bars indicate comparisons between two data sets assessed by a t-test. Significant differences are marked by an asterisk (all p-values $< 10^{-16}$). Effect size as measured by Cohen's d is indicated above bars. Increasing the dictionary size to 800 yielded a neglectable effect size of Cohen's $d < 0.017$ compared to $|\mathcal{B}_S| = 600$.

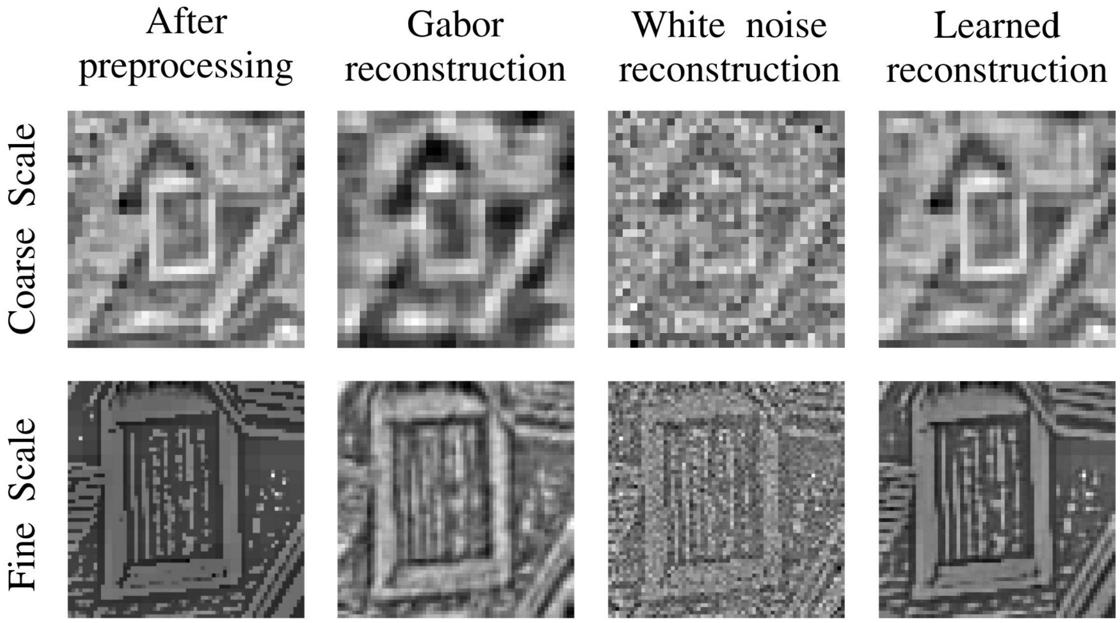

Figure S2. Input image reconstruction. Depicted are column wise from left to right the cropped, down-sampled and normalized input image for the coarse (top row) and fine scale (bottom row) sparse coders. Right to the preprocessed images are the respective images reconstructed with random Gabor wavelets at initialization time, the reconstructions with random white noise at initialization time and the images reconstructed with learned basis functions at the end of training when the basis functions were initialized with white noise. The agent improves in encoding and reconstructing the images over the coarse of training and can properly reconstruct the input images at the end of training. Regardless whether the agent's basis functions were initialized with random white noise or random Gabor wavelets, the reconstruction quality at the end of training is similar.

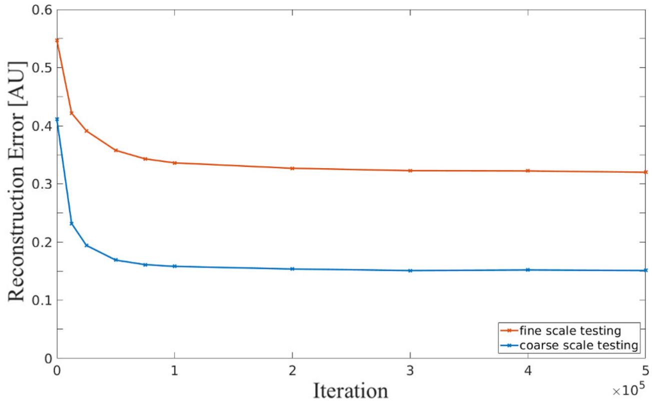

Figure S3. Reconstruction error of the sparse coding model in the testing procedure. The error is plotted in arbitrary units versus training time for the coarse scale (blue) and fine scale (red) sparse coder.
